# The Role of the SOX9/lncRNA ANXA2P2/miR-361-3p/SOX9 Regulatory Loop in Cervical Cancer Cell Growth and Resistance to Cisplatin

**DOI:** 10.3389/fonc.2021.784525

**Published:** 2022-01-10

**Authors:** Shasha He, Yeqian Feng, Wen Zou, Jingjing Wang, Guiyuan Li, Wei Xiong, Yangchun Xie, Jin-an Ma, Xianling Liu

**Affiliations:** ^1^ Department of Oncology, The Second Xiangya Hospital, Central South University, Changsha, China; ^2^ Cancer Research Institute of Central South University, Changsha, China

**Keywords:** cervical cancer, cisplatin (DDP), SOX9, miR-361-3p, lncRNA ANXA2P2

## Abstract

Cervical cancer is a highly prevalent female malignancy. Presently, cisplatin (DDP) is a first-line agent for cervical cancer chemotherapy. However, its curative effect is limited because of chemo-resistance. It has been previously reported that SOX9 targeted and activated oncogenic genes, enhancing cervical cancer cell resistance to DDP. The effects of the SOX9/lncRNA ANXA2P2/miR-361-3p/SOX9 regulatory loop on cervical cancer cell growth and resistance to DDP have been demonstrated. miR-361-3p expression was decreased in DDP-resistant cervical cancer cells and tissues. Moreover, miR-361-3p overexpression inhibited the growth of resistant cervical cancer cells and the resistance to DDP, whereas miR-361-3p inhibition exerted opposite effects. miR-361-3p inhibited SOX9 expression through binding; the effects of miR-361-3p inhibition were partially reversed by SOX9 knockdown. LncRNA ANXA2P2 expression was elevated in DDP-resistant cervical cancer cells and tissues. LncRNA ANXA2P2 inhibited miR-361-3p expression by binding, thereby upregulating SOX9. LncRNA ANXA2P2 knockdown inhibited DDP-resistant cervical cancer cell growth and resistance to DDP, whereas the effects of lncRNA ANXA2P2 knockdown were partially reversed by miR-361-3p inhibition. SOX9 expression was elevated in DDP-resistant cervical cancer cells and tissues, and SOX9 activated lncRNA ANXA2P2 transcription by binding. Collectively, SOX9, lncRNA ANXA2P2, and miR-361-3p form a regulatory loop, modulating DDP-resistant cervical cancer cell growth and response to DDP treatment.

## Introduction

Cervical cancer is a widespread female malignancy with high incidence and results in over 300,000 yearly fatalities worldwide (1, 2). Although human papillomavirus (HPV) infection has been regarded as a leading cause of cervical cancer (3–5), genetic factors might also affect the progression of HPV infection to cervical pre-cancer and cervical cancer (6). Presently, cisplatin (DDP) is a first-line agent for cervical cancer chemotherapy; however, its curative effect is limited because of a large portion of patients acquiring chemo-resistance (7). Therefore, the identification of new prognostic biomarkers would provide clinicians with potential therapeutic targets for personalized treatment regimens.

miRNA, a family of small endogenous RNA of 19–22 bp in length and devoid of protein-coding capacity, could result in the target mRNA degradation or translation inhibition through complete or incomplete complementarity to the 3’-untranslated region (3’-UTR) of target mRNA (8). For instance, our group reported miR-130a (9, 10) and miR-21 (11) modulating cervical cancer cell growth and resistance to DDP through targeting CTR1 or PTEN. There are more miRNAs that have been reported as multifaceted players regulating cancer aggressiveness and tumor microenvironment formation, such as miR-361. miR-361-3p is often reduced or lost in numerous kinds of malignancies and participates in tumor growth, epithelial–mesenchymal transition (EMT), distant migration, chemo- or radio-resistance, glycolysis, angiogenesis, and inflammation by inhibiting the expression of its target genes (12–16). Although Wang et al. (15) regarded miR-361-3p as an anti-tumor miRNA in cervical cancer-inhibiting cancer cell growth and invasion, its role, targets, and mechanism in cervical cancer resistance to DDP are still unclear.

Long non-coding RNAs (lncRNAs) are non-coding transcripts of lengths greater than 200 nucleotides. It lacks the potential to encode proteins but performs a variety of cellular functions (17), thereby exerting biological functions in tumor development and drug resistance (18, 19). Although lncRNAs could regulate gene expression in multiple ways, one of the common ways is the competing endogenous RNA (ceRNA) hypothesis, which states that lncRNAs serve as miRNA sponges, competing with miRNA target mRNAs and counteracting miRNA-mediated suppression on target mRNAs, also known as the competing endogenous RNA (ceRNA) mechanism. Through the ceRNA mechanism, lncRNAs participate in miRNA biological functions by rescuing miRNA targets’ expression (20–22). For instance, lncRNA GAS5 inhibited cisplatin-induced cervical cancer cell apoptosis by regulating the miR-21/STAT3 axis (23). In our previous study, it has also been demonstrated that lncRNA CASC2 serves as a ceRNA for miR-21, modulating cervical cancer cell resistance to DDP through targeting PTEN (11). Since the role of the transcriptional factor SOX9 in activating oncogenic miR-130a had already been reported (9), this study aims to search for lncRNAs that are potentially correlated with SOX9 and miR-361.

This study has confirmed the expression of miR-361-3p in DDP-resistant cervical cancer cell lines and tissues and analyzed the association of miR-361-3p expression with the survival of cervical cancer patients. miR-361-3p overexpression and inhibition were achieved in DDP-resistant or original cervical cancer cells, and the cellular effects of miR-361-3p were examined. Since SOX9 activates oncogenic miR-130a (9), the affinity of miR-361-3p to SOX9 and the inhibition of SOX9 expression were investigated. The dynamic effects of the miR-361-3p/SOX9 axis on DDP-resistant cervical cancer cells were examined. Next, lncRNAs associated with SOX9 and miR-361-3p were analyzed, and ANXA2P2 was selected. ANXA2P2 expression was examined in cell lines and tissues, and the predicted lncRNA ANXA2P2 binding to miR-361-3p was verified. The dynamic effects of the lncRNA ANXA2P2/miR-361-3p axis on DDP-resistant cervical cancer cells were examined. Finally, the predicted SOX9 binding to lncRNA ANXA2P2 was verified. Altogether, a novel regulatory axis modulating the growth and DDP resistance of cervical cancer cells has been identified.

## Materials and Methods

### Clinical Sampling

With the approval of the Ethics Committee of Central South University (ethic approval No. 2020542), 50 DDP-sensitive and DDP-resistant cervical cancer tissues were harvested from patients who underwent surgical resection or biopsy and combined radiotherapy and chemotherapy at the Second Xiangya Hospital. All patients had signed an informed consent approved by the Institutional Review Board. Samples were immediately transferred to a −80°C container. The clinical features of the patients are listed in **Table 1**. All the patients had received a combination of DDP-based chemotherapy and radiotherapy; after 2 cycles of therapy (24–26), patients showcasing progression disease (PD), recurrence, or metastasis in 6 months were classified as DDP-resistant.

**Table 1:** The clinical characteristics of cervical cancer patients.

Patients number	Age	FIGO stage	Histological subtype (SCC, ADC, other)	Sensitive to DDP
1	52	III C1p	SCC	Y
2	56	III C1p	SCC	Y
3	66	III C1r	SCC	Y
4	56	III b	SCC	Y
5	55	III C1r	SCC	Y
6	39	II A2	SCC	Y
7	67	II B	SCC	Y
8	47	II A2	ADC	Y
9	50	II B	SCC	Y
10	42	III C1p	SCC	Y
11	45	II B	SCC	Y
12	48	II B	SCC	Y
13	61	III C1r	SCC	Y
14	53	III B	SCC	Y
15	55	III C1r	SCC	Y
16	56	III B	SCC	Y
17	41	III C1r	ADC	Y
18	67	II B	SCC	Y
19	47	II B	SCC	Y
20	53	II B	SCC	Y
21	71	II B	SCC	Y
22	49	I B2	ADC	Y
23	52	II A2	SCC	Y
24	51	IV A	SCC	Y
25	53	II B	SCC	Y
26	52	III C2p	SCC+ADC	Y
27	65	III B	SCC	Y
28	57	III C1p	SCC	Y
29	55	III A	SCC+ADC+NETs	Y
30	57	II A2	SCC	Y
31	38	III C1p	SCC	Y
32	57	II A2	SCC	Y
33	54	III C1r	SCC	Y
34	55	IV A	SCC	Y
35	36	III C1p	ADC	Y
36	52	III C1p	SCC	Y
37	41	III C2r	SCC	Y
38	56	II B	SCC	Y
39	47	III C1p	SCC	Y
40	48	III C1p	SCC	Y
41	48	III C1r	SCC	Y
42	50	III B	SCC	Y
43	62	II B	SCC	Y
44	56	III C1r	SCC	Y
45	56	II B	SCC	Y
46	51	III B	SCC	Y
47	60	III C1r	SCC	Y
48	69	III B	ADC	Y
49	57	III B	Small cell carcinoma	Y
50	59	III B	SCC	Y
51	46	III B	SCC	N
51	51	II B	SCC	N
53	52	IV B	SCC	N
54	57	III C1p	SCC	N
55	43	IV B	ADC+NETs	N
56	56	III C2r	SCC	N
57	48	III C2r	SCC	N
58	42	II A2	SCC	N
59	51	I B2	SCC	N
60	39	III C1p	SCC+ADC	N
61	37	I B2	SCC	N
62	57	IV B	SCC+ADC	N
63	57	II B	SCC	N
63	51	I B3	ADC	N
65	68	II B	SCC	N
66	40	II B	SCC	N
67	56	IV A	SCC	N
68	45	II B	SCC	N
69	44	I B3	SCC	N
70	59	II B	ADC	N
71	54	I B3	SCC	N
72	45	III B	SCC	N
73	46	II B	ADC	N
74	36	II A1	ADC	N
75	51	II A2	SCC	N
76	56	II A1	SCC	N
77	39	II B	SCC	N
78	57	II B	SCC	N
79	51	I B2	ADC	N
80	47	II B	ADC	N
81	59	III C1r	SCC	N
82	70	II B	SCC	N
83	57	II B	SCC	N
84	56	IV A	SCC	N
85	49	III C2R	Sarcomas	N
86	51	III C2R	SCC	N
87	63	III C1p	SCC	N
88	54	III C2p	ADC	N
89	52	II B	SCC	N
90	59	III C1p	ADC	N
91	46	III C1r	Sarcomas	N
92	49	IV B	Small cell carcinoma	N
93	61	III B	ADC	N
94	69	II B	SCC	N
95	54	II A2	ADC	N
96	45	IV B	SCC	N
97	47	IV A	Small cell carcinoma	N
98	63	IV B	Sarcomas	N
99	56	IV A	SCC	N
100	52	II A2	ADC	N

SCC, squamous cell carcinoma; ADC, adenocarcinoma; NETs, Neuroendocrine carcinoma; Other, small cell carcinoma, sarcomas.

### Cell Lineages and Cell Culture

Caski (CRM-CRL-1550™) and HeLa (CCL-2™) cell lines were obtained from the American Type Culture Collection (ATCC; Manassas, VA, USA). HeLa cells were cultured in Eagle’s Minimum Essential Medium (Gibco; Waltham, MA, USA) added with 10% FBS (Invitrogen; Waltham, MA, USA). Caski cells were cultured in RPMI-1640 medium (Gibco) added to 10% FBS (Invitrogen). Both cell lines were cultured at 37°C in a CO_2_ saturation of 5%.

### Constructing DDP-Resistant Cervical Cancer Cell Lines

The cells were passaged by trypsinization, and the incremental and intermittent DDP treatment were combined to establish DDP-resistant Caski and HeLa cell lines. The initial DDP concentration in the culture medium was 0.01 μM and the cells were cultured in that concentration for 24 h. After removing DDP, cells were continually cultured until stable growth at under 0.01 μM DDP. DDP concentration was subsequently doubled, and the cycles were repeated until the highest concentration (8 μM) was reached. After about 10 months, DDP-resistant Caski and HeLa cell lines, Caski/DDP and HeLa/DDP, were obtained.

### Cell Transfection

Original Caski and HeLa cells, and DDP-resistant Caski/DDP and HeLa/DDP cells were transfected with miR‐361‐3p mimics, miR-361-3p inhibitor, the specific small interfering RNAs targeting SOX9 (siRNA1/2/3-SOX9), lncRNA ANXA2P2 (si-ANXA2P2), and the negative control (mimics NC, inhibitor NC, and si‐NC), respectively. The specific sequences are listed in **Table S1**. All the transfection vectors were synthesized and procured from GenePharma (Shanghai, China). Lipofectamine 3000 (Invitrogen) was applied for cell transfection for 48 h.

### Resistance Confirmation

Original Caski and HeLa cells, and DDP-resistant Caski/DDP and HeLa/DDP cells were cultured in 96-well plates. After 24 h of incubation, the cells were treated with 1, 2, 4, 8, 16, or 32 μg/ml DDP. After 48 h of incubation, the Cell Counting Kit-8 (CCK-8) agent was added to detect the absorbance of each well at 450 nm. Inhibition rate = (1 − A450 value of the administration group)/A450 value of the control group × 100%. The half-maximal inhibitory concentration (IC50) calculator software was used to calculate the drug concentration required to inhibit 50% of the cell growth.

### CCK-8 Assay

Original Caski and HeLa cells, and DDP-resistant Caski/DDP and HeLa/DDP cells in the logarithmic growth phase were inoculated in 96-well plates at a density of 2 × 10^3^ cells/well. After undergoing culture for 24 h, the cells were treated and/or transfected, accordingly, and incubated. At the end of the incubation, 10 μl of CCK-8 agent was added into the wells and incubated for 2 h at 37°C. The absorbance was subsequently measured at 450 nm.

### qRT-PCR

Total RNA was extracted from cultured cells using the Trizol reagent (Invitrogen). The expression levels of miRNA and mRNA were measured using a SYBR Green qPCR assay (Takara, Dalian, China) (27). The expression of GAPDH (reference for mRNA determination) or RNU6B (reference for miRNA determination) served as an endogenous control, and the specific primers used are presented in **Table S1**. The 2^−ΔCT^ method was applied for data processing.

### Western Blot

After cell lysis in RAPI protein lysate (Beyotime, Shanghai, China) to collect total protein, quantification of the total protein was accomplished *via* Pierce BCA Protein Assay Kit (Pierce, Rockford, IL, USA). Protein separation was conducted by sodium dodecyl sulfate-polyacrylamide gel electrophoresis (SDS-PAGE, 200 mA, 120 min). The proteins were subsequently electro-blotted onto a PVDF membrane (Merck Millipore, Billerica, MA, USA). The membrane was then immersed in Tris Buffered Saline Tween (TBST) with 5% skim milk for 1 h at room temperature. The mixture was cultivated overnight with primary antibodies, including anti-SOX9 (1:1000, Cat# ab185966, Abcam, Cambridge, MA, USA) at 4°C. After having been washed thrice with TBST buffer, the HRP-linked antibody goat anti-rabbit IgG (1:1,000, ab6721, Abcam) was added, followed by incubation at room temperature on a shaker for 1.5 h. After TBST-wash in triplicate, the blots were finally visualized under the ECL chemiluminescence system, with their integral optical density analyzed using Lab Works4.5 software. β-actin was deemed the control for SOX9 protein.

### Colony Formation Assay

DDP-resistant Caski/DDP and HeLa/DDP cells were incubated in 6‐well plates at a density of 5 × 10^3^ cells/well. The cells were allowed to grow for 2 weeks and colonies were subsequently fixed with 4% paraformaldehyde for 30 min and were dyed in crystal violet (Sigma‐Aldrich; St. Louis, MO, USA) for 15 min. The visible colonies were counted manually under a microscope (Olympus, Tokyo, Japan).

### Dual-Luciferase Reporter Assay

Wild- and mutant-type SOX9 and ANXA2P2 luciferase reporter vectors were constructed by cloning SOX9 3’-UTR or ANXA2P2 fragment to psiCheck-2 vector (Promega, Madison, WI, USA) or inducing a mutation into the predicted miR-361-3p binding site. Reporter vectors were named wt-SOX9, wt-ANXA2P2, mut-SOX9, or mut-ANXA2P2. Reporter vectors were co-transfected in 293T cells with miR-361-3p mimics or miR-361-3p inhibitor. Luciferase alterations were then determined in a Dual-luciferase Reporter Assay System (Promega).

The lncRNA-ANXA2P2 promoter region fragment (contains 2,048 to 2,056 bp) construct was amplified from genomic DNA of Caski/DDP and HeLa/DDP cells. The wt and mutated ANXA2P2 promoter constructs were cloned into the pGL3-basic reporter gene vector and verified through sequencing. To determine the activities of ANXA2P2 promoter constructs, Caski/DDP and HeLa/DDP cells were transfected by Lipofectamine 2000 in six-well plates followed by dual-luciferase reporter assays (Promega). The specific sequences are listed in **Table S1**.

### Chromatin Immunoprecipitation Assay

ChIP assays were performed following the Upstate Chromatin Immunoprecipitation Protocol (www.upstate.com) and the aforementioned method (28) using anti-IgG and anti-SOX9. The immune complexes were eluted from the beads by addition of elution buffer (1% SDS, 0.1 M NaHCO_3_), vortexing for 30 s, and mixing at RT for 15 min and centrifugation to collect the eluates. Elution was performed twice. The eluates were combined and followed by the addition of sodium chloride (0.33 M final concentration) to reverse crosslinking by incubation overnight at 65°C. DNA was isolated and purified by phenol:chloroform extraction and isopropanol precipitation and subsequently used for PCR with primers designed to be flanking the putative SOX9-binding site of the ANXA2P2 promoter.

### Xenograft Mice Assay *In Vivo*

Sterile conditions were maintained through the lifetime of twenty-four male BALB/c nude mice (4 weeks old). The approval of xenograft *in vivo* assay was obtained from Second Xiangya Hospital. Firstly, antagomiR-NC and antagomiR-361-3p were synthesized by BGI (Shenzhen, China). The knockdown of ANXA2P2 (Lv-sh-ANXA2P2) or miR-361-3p (antagomiR-361-3p) was performed using MISSION^®^ shRNA lentiviral particles (Sigma-Aldrich), which have been designed to inhibit the production of ANXA2P2 or miR-361-3p in CaSki/DDP and HeLa/DDP cells. Subsequently, Lv-sh-ANXA2P2 or antagomiR-361-3p transfected CaSki/DDP and HeLa/DDP cells (1×10^6^) were subcutaneously injected into the armpit of nude mice, respectively. A caliper was used to measure tumor volume following the length×width^2^/2 formula. The average volume of the tumor was measured thrice every 3 days. At the termination of the experiment (the 22nd day), mice were sacrificed and the tumor was excised from each mouse to measure the average volume and weight.

### Hematoxylin and Eosin Staining for Histological Analysis

Tumors were weighed and excised immediately after sacrificing the mice. The tumor tissues were fixed in 10% neutral-buffered formalin overnight. The sections were stained with hematoxylin and eosin (H&E) to observe morphological changes.

### Immunohistochemistry Staining

The tumor tissues were used for immunohistochemical staining to detect E-cadherin, N-cadherin, Vimentin, cleaved caspase 3, and SOX9 levels. The tissues were embedded in paraffin, and the cross-sections were then deparaffinized and rehydrated through graded alcohols and washed in PBS twice for 10 min. The slices were incubated for a night with rabbit polyclonal primary antibody of E-cadherin (Cat# ab1416, Abcam), N-cadherin (Cat# ab76011, Abcam), Vimentin (Cat# ab92547, Abcam), cleaved caspase 3 (Cat# ab32351, Abcam), and SOX9 (Cat# ab185966, Abcam). The sections were subsequently incubated with 45 µl of secondary antibody horseradish peroxidase-conjugated goat anti-rabbit IgG H&L (HRP) (Cat# ab6721, 1/1000, Abcam) at 37°C for 30 min. The slices were dyed with 3,3’-diaminobenzidine (DAB) working solution for 3 min, then washed in water for 10 min, and counterstained with hematoxylin. After rewashing the slices in water for 10 min, the cross-sections were dehydrated and cleared. The slices were finally observed and photographed with a light microscope.

### Data Processing and Statistical Analysis

The data were analyzed with the GraphPad software. The measurement data were expressed as mean ± standard deviation (SD). Among-group and intra-group data comparisons were performed with the ANOVA followed by Tukey’s post-hoc test or Student’s *t*-tests. *p* < 0.05 indicated a statistically significant difference.

## Results

### miR-361-3p Underexpression of in DDP-Resistant Cervical Cancer Cells

**Figure 1:**
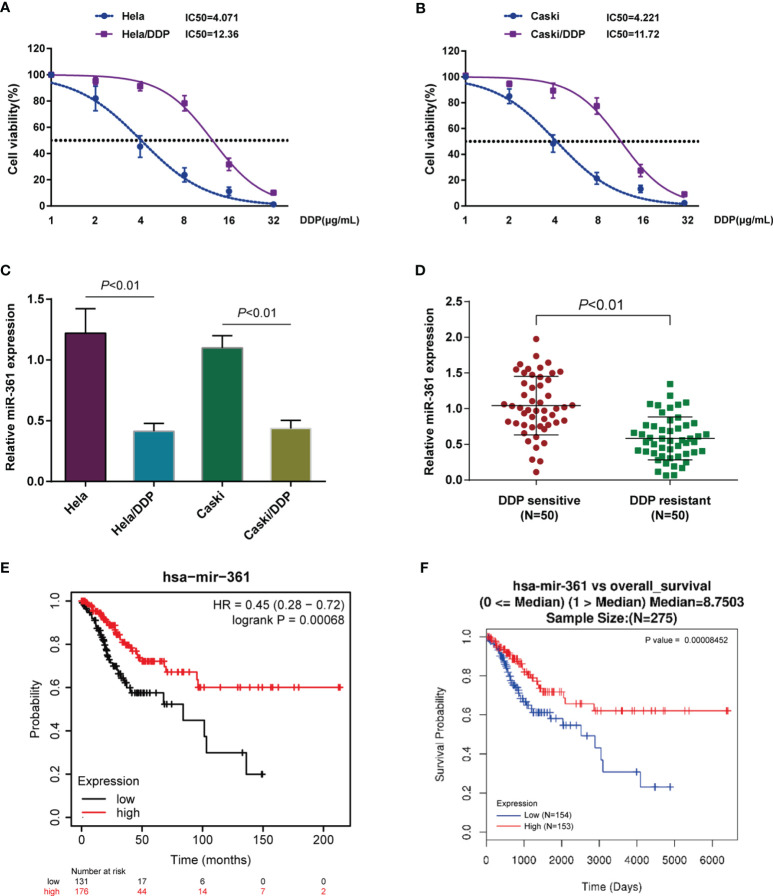
Underexpression of miR-361-3p in cisplatin (DDP)-resistant cervical cancer cells. **(A, B)** Original Caski and HeLa cells and DDP-resistant Caski/DDP and HeLa/DDP cells were exposed to 1, 2, 4, 8, 16, or 32 μg/ml DDP and the cell viability was determined using CCK-8 assay. IC50 values were calculated and shown. **(C)** miR-361-3p expression in original Caski and HeLa cells and DDP-resistant Caski/DDP and HeLa/DDP cells was determined using qRT-PCR. **(D)** miR-361-3p expression in DDP-sensitive and DDP-resistant cervical cancer tissues (*n* = 50) was determined using qRT-PCR. **(E)** Correlation of miR-361-3p expression with the survival probability of patients with cervical cancer was analyzed using the online tool Kaplan–Meier plotter based on TCGA data from Pan-Cancer Atlas. **(F)** A Cox proportional hazard regression model was used to analyze to correlation of miR-361-3p expression with the overall survival in patients with cervical cancer using the linkedOmics website (http://www.linkedomics.org/login.php) based on TCGA-CESC data.

Abnormally expressed miRNAs were identified through bioinformatics analysis in 167 chemotherapy complete-response patients compared with 27 progressive disease patients based on the TCGA-CESC cervical cancer data and were visualized in a volcano plot (**Figure S1A**) and heatmap (**Figure S1B**). Five significantly downregulated miRNAs (hsa-miR-1269a, hsa-miR-16-2-3p, hsa-miR-660-5p, hsa-miR-361-3p, and hsa-miR-616-5p) were found using the threshold of false-positive rate less than 0.05 and |logFC| > 0.4 (**Table S2**). To investigate the expressions of those miRNAs in cervical cancer DDP resistance, DDP-resistant cervical cancer cell lines based on Caski and HeLa cells were firstly analyzed. To confirm the DDP resistance, original Caski and HeLa cells and DDP-resistant Caski/DDP and HeLa/DDP cells were exposed to 1, 2, 4, 8, 16, or 32 μg/ml DDP and the IC50 values were evaluated by assessing cell viability. **Figures 1A, B** show that the IC50 values for Caski/DDP and HeLa/DDP were significantly elevated compared to those for the original Caski and HeLa cells, from 4.071 to 12.36 (HeLa/DDP) and 4.221 to 11.72 (Caski/DDP). The expressions of those miRNAs in cervical cancer DDP resistance cells and normal cervical cancer cells were subsequently detected (**Figure S2C**). Among them, in Caski/DDP and HeLa/DDP cells, miR-361-3p expression was the most significantly downregulated compared to those of the original Caski and HeLa cells (**Figure 1C** and **Figure S2C**). Consistently, miR-361-3p expression was decreased in DDP-resistant cervical cancer tissues in comparison to those in DDP-sensitive samples (**Figure 1D**). Moreover, miR-361-3p expression was decreased in progressive disease patients when compared with chemotherapy complete response patients based on TCGA-CESC data (**Figure S2D**). Hence, miR-361-3p was selected for further investigations.

Moreover, the correlation of miR-361-3p expression with the survival probability of cervical cancer patients was analyzed through the Kaplan–Meier plotter (https://kmplot.com/analysis/) based on TCGA data from Pan-Cancer Atlas; as illustrated in **Figure 1E**, elevated miR-361-3p expression was associated with higher survival probability. Moreover, a Cox proportional hazard regression model indicated that higher miR-361-3p expression predicted better overall survival in cervical cancer patients using the linkedOmics website (http://www.linkedomics.org/login.php) based on the TCGA-CESC data (**Figure 1F**).

### Overexpressing miR-361-3p Could Re-Sensitize DDP-Resistant Cervical Cancer Cells to DDP

**Figure 2:**
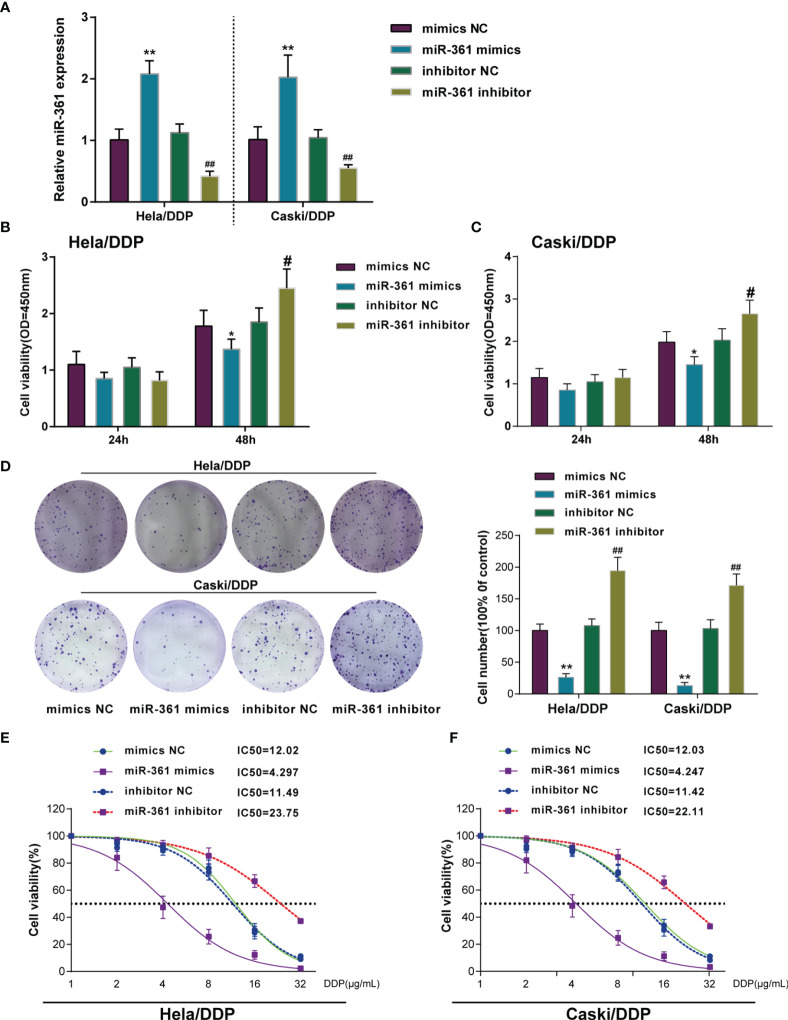
Overexpressing miR-361-3p could re-sensitize DDP-resistant cervical cancer cells to DDP. **(A)** miR-361-3p overexpression or inhibition was achieved in DDP-resistant cervical cancer cells by transfecting miR-361-3p mimics or inhibitor. miR-361-3p overexpression or inhibition was confirmed in target cells by qRT-PCR. **(B, C)** DDP-resistant cervical cancer cells were transfected with miR-361-3p mimics or inhibitor and examined for cell viability by CCK-8 assay. **(D)** DDP-resistant cervical cancer cells were transfected with miR-361-3p mimics or inhibitor and examined for colony formation capacity by colony formation assay. **(E, F)** DDP-resistant cervical cancer cells were transfected with miR-361-3p mimics or inhibitor, exposed to 1, 2, 4, 8, 16, or 32 μg/ml DDP, and examined for cell viability by CCK-8 assay. IC50 values were calculated and shown. *p < 0.05, **p < 0.01, ^#^ *p* < 0.05, ^##^ *p* < 0.01.

Since miR-361-3p is significantly downregulated in DDP-resistant cervical cancer cells and tissues, miR-361-3p overexpression or inhibition was achieved in DDP-resistant Caski/DDP and HeLa/DDP cells, and the cellular effects of miR-361-3p overexpression and inhibition were investigated. miR-361-3p overexpression or inhibition was confirmed in target cells by qRT-PCR (**Figure 2A**). The cell viability of miR-361-overexpressing cells (**Figures 2B, C**) and colony formation capacity was inhibited in both cell lines (**Figure 2D**), whereas miR-361-3p inhibition exerted opposite effects on both cell lines (**Figures 2B, D**). DDP-resistant cervical cancer cells were subsequently transfected with miR-361-3p mimics or inhibitor, exposed to 1, 2, 4, 8, 16, or 32 μg/ml DDP, and examined for cell viability and IC50 values; miR-361-3p overexpression significantly lowered the IC50 values for both cell lines, whereas miR-361-3p inhibition further elevated the IC50 values (**Figures 2E, F**).

### SOX9 Is Upregulated in DDP-Resistant Cervical Cancer and a Direct Target of miR-361

**Figure 3:**
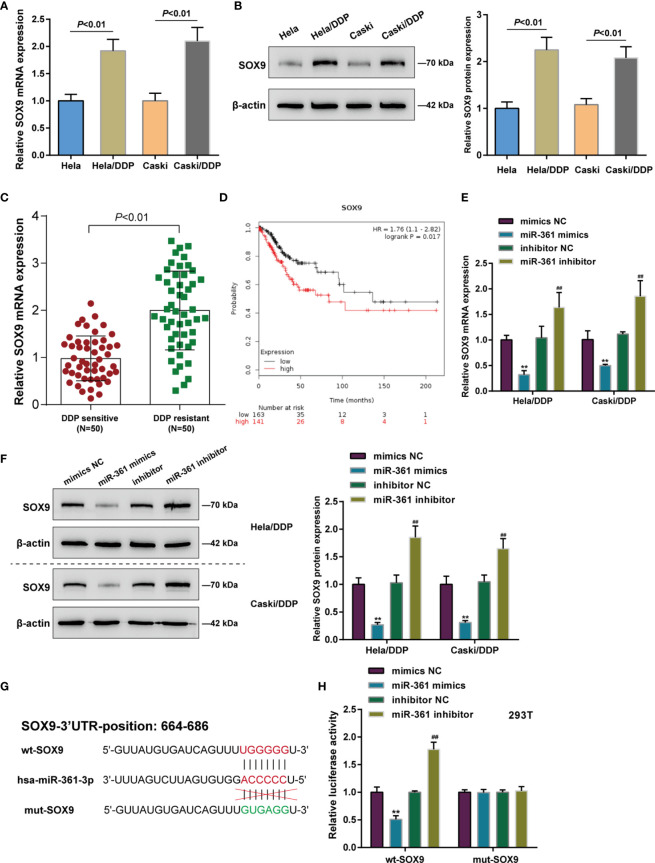
SOX9 is upregulated in DDP-resistant cervical cancer and a direct target of miR-361-3p. **(A, B)** SOX9 mRNA **(A)** and protein **(B)** expression in original Caski and HeLa cells and DDP-resistant Caski/DDP and HeLa/DDP cells was determined using qRT-PCR and Western blot assays. **(C)** SOX9 expression in DDP-sensitive and DDP-resistant cervical cancer tissues (*n* = 50) was determined using qRT-PCR. **(D)** Correlation of SOX9 expression with the survival probability of patients with cervical cancer was analyzed using the online tool Kaplan–Meier plotter based on TCGA data from Pan-Cancer Atlas. **(E, F)** DDP-resistant cervical cancer cells were transfected with miR-361-3p mimics or inhibitor and examined for SOX9 mRNA **(E)** and protein **(F)** expression by qRT-PCR and Western blot assays. **(G)** Wild- and mutant-type SOX9 luciferase reporter vectors were constructed by cloning SOX9 3’-UTR to psiCheck-2 vector or inducing mutation to the predicted miR-361-3p binding site. **(H)** Wild- and mutant-type SOX9 luciferase reporter vectors were co-transfected to 293T cells with miR-361-3p mimics or inhibitor. The luciferase activity was determined using Dual-luciferase Reporter Assay System. **p < 0.01, ^##^ *p* < 0.01.

SOX9 targeting oncogenic miR-130a to activate its expression and therefore affecting cervical cancer chemo-resistance to DDP through the miR-130a/CTR1 axis has been previously recognized (9). Here, the miRWalk online tool predicted SOX9 as a direct downstream target of miR-361-3p. Next, the role of SOX9 and its correlation with miR-361-3p was investigated. Differing from miR-361, SOX9 mRNA and protein expression was significantly upregulated in DDP-resistant Caski/DDP and HeLa/DDP cells compared with that in original Caski and HeLa cells (**Figures 3A, B**), and upregulated in DDP-resistant cervical cancer tissues in comparison with non-resistant samples (**Figure 3C**). Based on TCGA-CESC data, higher SOX9 expression was associated with lower survival likelihood of cervical cancer patients, as revealed by the analysis conducted by the Kaplan–Meier plotter (**Figure 3D**).

As for the predicted miR-361-3p regulation of SOX9, DDP-resistant cervical cancer cells were transfected with miR-361-3p mimics or inhibitor, and SOX9 mRNA and protein expression was examined; **Figures 3E, F** depict that miR-361-3p negatively regulated SOX9 mRNA and protein expression in both Caski/DDP and HeLa/DDP cells. As for predicted miR-361-3p binding to SOX9, wild- and mutant-type SOX9 luciferase reporter vectors were constructed by cloning SOX9 3’-UTR to psiCheck-2 vector or inducing mutation to the predicted miR-361-3p binding site (**Figure 3G**). These vectors were co-transfected to 293T cells with miR-361-3p mimics or inhibitor. When co-transfected with wt-SOX9, miR-361-3p overexpression was inhibited, whereas miR-361-3p inhibition enhanced the luciferase activity of wt-SOX9; miR-361-3p failed to alter the luciferase activity in cells co-transfected with mut-SOX9 (**Figure 3H**).

### Dynamic Effects of the miR-361-3p/SOX9 Axis on DDP-Resistant Cervical Cancer Cells

**Figure 4:**
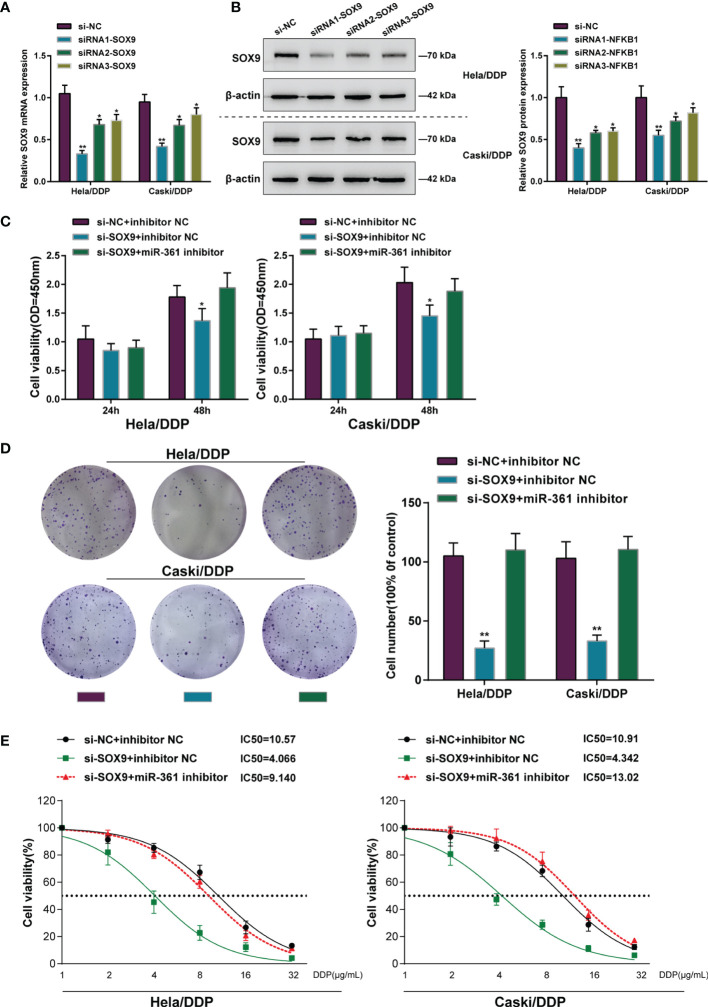
Dynamic effects of the miR-361-3p/SOX9 axis on DDP-resistant cervical cancer cells. **(A, B)** SOX9 knockdown was achieved in DDP-resistant cervical cancer cells by transfecting small interfering RNA against SOX9 (siRNA1/2/3-SOX9). SOX9 knockdown was confirmed in target cells by qRT-PCR **(A)** and Western blot **(B)** assays. **(C, D)** Then, DDP-resistant cervical cancer cells were co-transfected with si-SOX9 and miR-361-3p inhibitor and examined for cell viability by CCK-8 assay **(C)** and colony formation capacity by colony formation assay **(D)**. **(E)** DDP-resistant cervical cancer cells were co-transfected with si-SOX9 and miR-361-3p inhibitor, exposed to 1, 2, 4, 8, 16, or 32 μg/ml DDP, and examined for cell viability by CCK-8 assay. IC50 values were calculated and shown. *p < 0.05, **p < 0.01.

After confirming miR-361-3p regulation of SOX9, the dynamic effects of the miR-361-3p/SOX9 axis on cervical cancer cell resistance to DDP was subsequently investigated. SOX9 knockdown in DDP-resistant cervical cancer cells was achieved by transfecting small interfering RNA against SOX9 (siRNA1/2/3-SOX9). SOX9 knockdown was confirmed in target cells by qRT-PCR and Western blot (**Figures 4A, B**). DDP-resistant cervical cancer cells were then co-transfected with si-SOX9 and miR-361-3p inhibitor and examined for cell phenotypes. SOX9 knockdown significantly inhibited viability and colony formation capacity, and partially attenuated the effects of miR-361-3p inhibition (**Figures 4C, D**). DDP-resistant cervical cancer cells were subsequently co-transfected with si-SOX9 and miR-361-3p inhibitor, exposed to 1, 2, 4, 8, 16, or 32 μg/ml DDP, and examined for cell viability and IC50 values. SOX9 knockdown significantly lowered the IC50 values of both cell lines, and partially reversed the effects of miR-361-3p inhibition on DDP-resistant cervical cancer cells’ response to DDP treatment (**Figure 4E**).

### LncRNA ANXA2P2 Regulates miR-361-3p and SOX9 Expression

**Figure 5:**
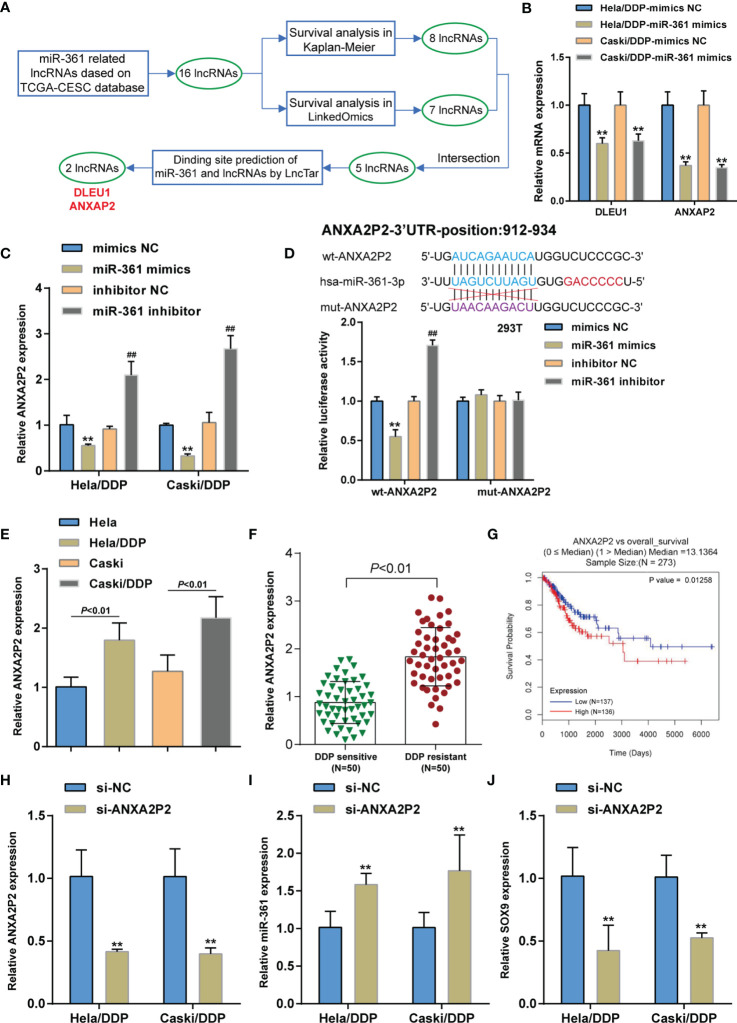
LncRNA ANXA2P2 regulates miR-361-3p and SOX9 expression. **(A)** Two lncRNAs (DLEU1 and ANXAP2) that targeted miR-361-3p in cervical cancer were screened based on the TCGA-CESC database by correlation analysis and Kaplan–Meier plotter and LinkedOmics database by survival analysis. **(B)** DDP-resistant cervical cancer cells were transfected with miR-361-3p mimics and examined for DLEU1 and ANXAP2 expression by qRT-PCR. **(C)** DDP-resistant cervical cancer cells were transfected with miR-361-3p mimics or inhibitors and examined for ANXA2P2 expression by qRT-PCR. **(D)** Wild- and mutant-type ANXA2P2 luciferase reporter vectors were constructed by cloning ANXA2P2 fragment to psiCheck-2 vector or inducing mutation to the predicted miR-361-3p binding site. Luciferase reporter vectors were co-transfected to 293T cells with miR-361-3p mimics or inhibitor. The luciferase activity was determined using the Dual-luciferase Reporter Assay System. **(E)** ANXA2P2 expression in original Caski and HeLa cells and DDP-resistant Caski/DDP and HeLa/DDP cells was determined using qRT-PCR. **(F)** ANXA2P2 expression in DDP-sensitive and DDP-resistant cervical cancer tissues (*n* = 50) was determined using qRT-PCR. **(G)** A Cox proportional hazard regression model was used to analyze the correlation of ANXA2P2 expression with the overall survival in patients with cervical cancer using the linkedOmics website based on TCGA-CESC data. **(H)** ANXA2P2 knockdown was achieved in DDP-resistant cervical cancer cells by transfecting small interfering RNA against ANXA2P2 (si-ANXA2P2). ANXA2P2 knockdown was confirmed by qRT-PCR. **(I, J)** DDP-resistant cervical cancer cells were transfected with si-ANXA2P2 or si-NC and examined for the expression of miR-361-3p and SOX9 by qRT-PCR. **p < 0.01, ^##^ *p* < 0.01.

As previously reviewed, lncRNAs commonly act as ceRNA for miRNAs, leading to miRNA inhibition and the upregulation of miRNA targets (20, 29–31). Considering the aberrant miR-361-3p downregulation in DDP-resistant cervical cancer, lncRNAs that might significantly negatively correlate with the expression of miR-361-3p were searched for on the TCGA-CESC database and 16 related ncRNAs were found (**Table S3**). The association of these lncRNAs with the overall survival of cervical cancer patients was subsequently predicted by the Kaplan–Meier plotter (**Figure S2A**) and LinkedOmics (http://www.linkedomics.org/login.php) (**Figure S2B**) database and five lncRNAs (DLEU1, SNORA8, UBE2Q2P1, SCARNA8, and ANXA2P2) were obtained. Finally, the LncTar website (http://www.cuilab.cn/lnctar) was used to predict the potential binding sites, in which DLEU1 and ANXAP2 may bind to miR-361 (**Figure 5A**). Among two lncRNAs, the ANXAP2 mRNA expression level was the lowest in the miR-361-3p mimics group in two DDP-resistant cervical cancer cells (**Figure 5B**). Moreover, higher ANXA2P2 expression could be potentially associated with glioma progression or poorer prognosis (32–35). Hence, lncRNA ANXAP2 was selected for further investigations. In DDP-resistant cervical cancer cells, miR-361-3p overexpression was downregulated, whereas miR-361-3p inhibition upregulated ANXA2P2 expression (**Figure 5C**). The Dual-LUC analysis was subsequently performed to validate the predicted ANXA2P2 binding to miR-361. Wild- and mutant-type ANXA2P2 luciferase reporter vectors were constructed and co-transfected to 293T cells with miR-361-3p mimics or inhibitor. When co-transfected with wt-ANXA2P2, miR-361-3p overexpression was remarkably inhibited, whereas miR-361-3p inhibition elevated wt-ANXA2P2 luciferase activity; when co-transfected with mut-ANXA2P2, miR-361-3p failed to alter luciferase activity (**Figure 5D**). Similar to SOX9, ANXA2P2 expression was dramatically upregulated in DDP-resistant Caski/DDP and HeLa/DDP cells compared with that in original Caski and HeLa cells (**Figure 5E**), and upregulated in DDP-resistant cervical cancer tissues compared to those in non-resistant samples (**Figure 5F**). The correlation of ANXA2P2 expression with the overall survival in patients with cervical cancer was subsequently analyzed using a Cox proportional hazard regression model on the linkedOmics website based on TCGA-CESC data. As illustrated in **Figure 5G**, higher ANXA2P2 expression predicted poorer overall survival in patients with cervical cancer. To confirm ANXA2P2 regulation of miR-361-3p and SOX9, respectively, ANXA2P2 knockdown was achieved in DDP-resistant cervical cancer cells by transfecting small interfering RNA against ANXA2P2 (si-ANXA2P2). ANXA2P2 knockdown was confirmed by qRT-PCR (**Figure 5H**). In DDP-resistant cervical cancer cells, ANXA2P2 knockdown upregulated miR-361-3p expression but downregulated SOX9 expression (**Figures 5I–J**). Thus, ANXA2P2 was found to target miR-361, inhibit miR-361-3p expression, and promote SOX9 expression.

### The SOX9/lncRNA ANXA2P2/miR-361-3p/SOX9 Regulatory Loop Modulates Cervical Cancer Cell Resistance to DDP

**Figure 6:**
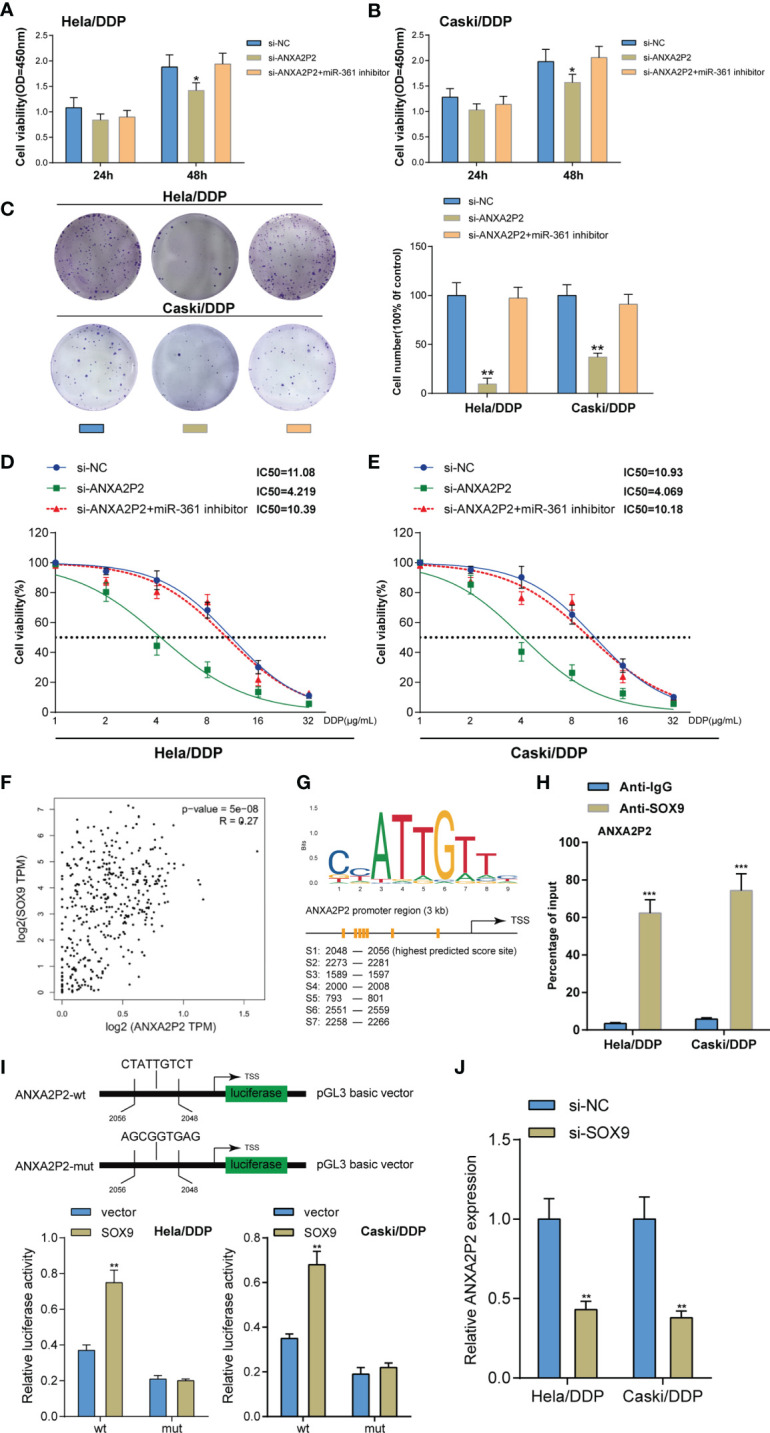
Dynamic effects of the ANXA2P2/miR-361-3p axis on DDP-resistant cervical cancer cells. DDP-resistant cervical cancer cells were co-transfected with si-ANXA2P2 and miR-361-3p inhibitor and examined for cell viability by CCK-8 assay **(A, B)** and colony formation capacity by colony formation assay **(C)**. **(D, E)** DDP-resistant cervical cancer cells were co-transfected with si-ANXA2P2 and miR-361-3p inhibitor, exposed to 1, 2, 4, 8, 16, or 32 μg/ml DDP, and examined for cell viability by CCK-8 assay. IC50 values were calculated and shown. **(F)** The Cox linear regression analysis on the correlation between SOX9 and ANXA2P2 using 304 cases of cervical squamous carcinoma samples from the TCGA-CESC database. **(G)** Jaspar predicted the possible binding sites between SOX9 and the ANXA2P2 promoter. **(H)** ChIP assay was performed using anti-IgG (negative control) or anti-SOX9. The levels of the ANXA2P2 promoter in the immunoprecipitate of anti-IgG and anti-SOX9 were determined using qRT-PCR. **(I)** The binding site of SOX9 was at the ANXA2P2 promoter region 2,048 to 2,056 bp and the predicted binding site was mutated (ANXA2P2-mut). Then, the luciferase activity of ANXA2P2 was detected using Dual-luciferase report assay in HeLa/DDP and Caski/DDP cells after transfection of the ANXA2P2-wt or ANXA2P2-mut alone or together with the SOX9 expression vector. **(J)** HeLa/DDP and Caski/DDP cells were co-transfected with si-SOX9 and examined for ANXA2P2 expression by qRT-PCR assay *p < 0.05, **p < 0.01, ***p < 0.001.

After confirming ANXA2P2 targeting and inhibiting miR-361, the dynamic effects of ANXA2P2 and miR-361-3p on DDP-resistant cervical cancer cells were detected. Target cells were co-transfected with si-ANXA2P2 and miR-361-3p inhibitor and examined for cell phenotypes. ANXA2P2 knockdown inhibited cell viability and colony formation capacity, whereas miR-361-3p inhibition partially reversed the effects of ANXA2P2 knockdown (**Figures 6A**–**C**). Next, DDP-resistant cervical cancer cells were co-transfected with si-ANXA2P2 and miR-361-3p inhibitor, exposed to 1, 2, 4, 8, 16, or 32 μg/ml DDP, and examined for cell viability. As illustrated in **Figures 6D, E**, ANXA2P2 knockdown significantly lowered the IC50 values of both cell lines, whereas miR-361-3p inhibition partially elevated the IC50 values. These data indicate that miR-361-3p could partially reverse the effects of ANXA2P2 on DDP-resistant cervical cancer cells.

SOX9 has been recognized as an oncogenic gene enhancing cervical cancer resistance to DDP (9). Firstly, the Cox linear regression analysis using 304 cases of cervical squamous carcinoma samples from the TCGA-CESC database demonstrated the positive correlation between SOX9 and ANXA2P2 (**Figure 6F**). It was then verified whether the ANXA2P2 promoter region obtained a response element for SOX9; ChIP-Atlas database (**Table 2**) and JASPAR tool (**Table 3**) indicated the possible binding between SOX9 and ANXA2P2, and the highest predicting scores of the potential binding site were selected for further investigation (**Figure 6G**). Thus, a ChIP assay was performed using anti-IgG (negative control) and anti-SOX9 to evaluate the levels of the ANXA2P2 promoter in the immunoprecipitate of anti-IgG and anti-SOX9. **Figure 6H** demonstrated that the levels of the ANXA2P2 promoter in the immunoprecipitate of anti-SOX9 were sharply elevated compared with those in the immunoprecipitate of anti-IgG in DDP-resistant Caski/DDP and HeLa/DDP cells. To further confirm the direct interaction between SOX9 and the ANXA2P2 promoter, the predicted SOX9-binding site was mutated at region 2,048 to 2,056 bp of the ANXA2P2 promoter (ANXA2P2-mut) (**Figure 6I**). Then, the wt-ANXA2P2 and mut-ANXA2P2 constructs were transfected alone or with the SOX9 expression vector into Caski/DDP and HeLa/DDP cells. The results of dual-luciferase reporter assays demonstrated that the promoter activity of wt-ANXA2P2 was markedly increased after transfection with the SOX9 expression plasmid. However, no changes were observed in wt-ANXA2P2 (**Figure 6I**). As displayed in **Figure 6J**, SOX9 inhibition dramatically inhibited ANXA2P2 expression in DDP-resistant cervical cancer cells.

**Table 2:** ChIP-Atlas database indicated the possible binding between SOX9 and ANXA2P2.

ID	Antigen class	Antigen	Cell class	Cell	Number of peaks	Overlaps/My data	Overlaps/Control
SRX092576	TFs and others	SOX9	Uterus	HeLa	1,546	0/0	1,412/18,550
SRX190209	TFs and others	SOX9	Uterus	ECC-1	14,363	0/0	7,631/18,550

**Table 3:** JASPAR indicated the possible binding between SOX9 and ANXA2P2.

Matrix ID	Name	Score	Relative score	Sequence ID	Start	End
MA0077.1	SOX9	9.41982	0.89741736324	ENST00000435128|ANXA2P2	2048	2056
MA0077.1	SOX9	8.65968	0.87559606993	ENST00000435128|ANXA2P2	2273	2281
MA0077.1	SOX9	8.50177	0.87106297128	ENST00000435128|ANXA2P2	1589	1597
MA0077.1	SOX9	7.90428	0.85391074494	ENST00000435128|ANXA2P2	2000	2008
MA0077.1	SOX9	7.8075	0.85113253148	ENST00000435128|ANXA2P2	793	801
MA0077.1	SOX9	7.2733	0.83579708994	ENST00000435128|ANXA2P2	2551	2559
MA0077.1	SOX9	7.07459	0.83009293624	ENST00000435128|ANXA2P2	2258	2266

### Dynamic Effects of the ANXA2P2/miR-361-3p Axis on the Growth of Xenograft Formed by DDP-Resistant Cervical Cancer Cells in Nude Mice

**Figure 7:**
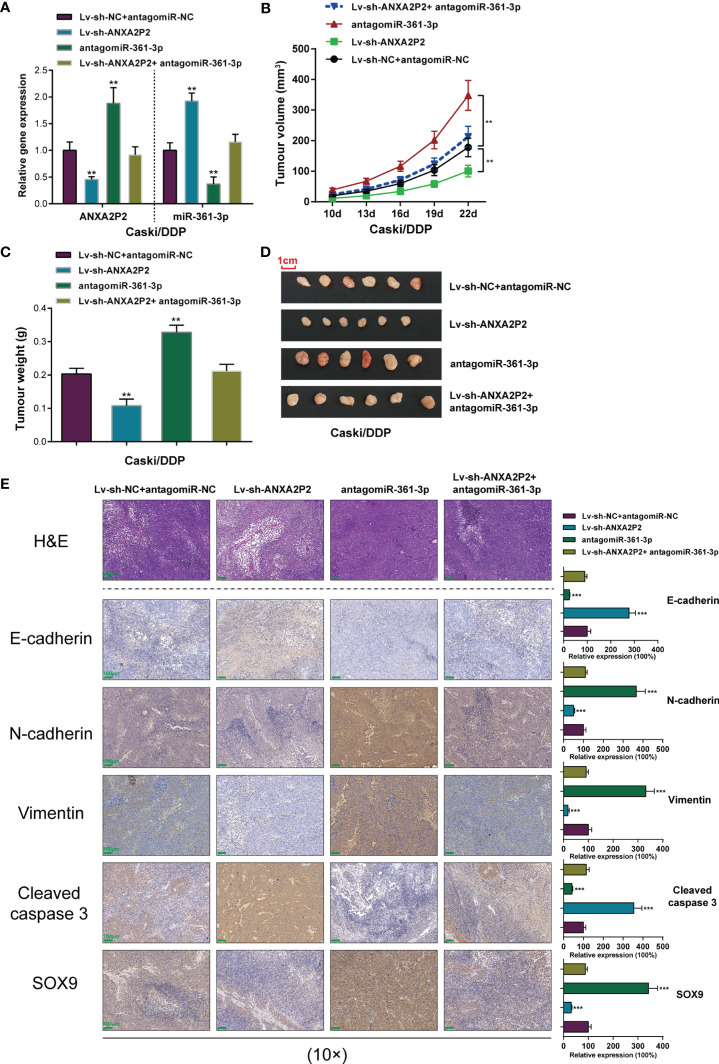
Effects of the ANXA2P2/miR-361-3p axis on the growth of xenograft formed by DDP-resistant cervical cancer cells in nude mice. Caski/DDP cells pre-transfected with lentivirus-mediated ANXA2P2 or miR-361-3p knockdown or negative control were subcutaneously injected into the armpit of nude mice, respectively. **(A)** ANXA2P2 or miR-361-3p knockdown was achieved in DDP-resistant cervical cancer cells by transfecting Lv-sh-ANXA2P2 or antagomiR-361-3p. The ANXA2P2 and miR-361-3p expressions were confirmed in target cells by qRT-PCR. **(B)** Tumor volumes of nude mice were measured every 3 days from the 10th day after injection. **(C)** Tumors were removed from nude mice 22 days after injection. On the 25th day, the tumor weights in different groups were detected. **(D)** Picture of mouse tumors illustrated that knockdown of ANXA2P2 significantly reduced tumor size, while miR-361-3p inhibition promoted tumor size. **(E)** The histopathological characteristics of the tumors were examined by hematoxylin and eosin (H&E) staining, and the tumor metastasis proteins (E-cadherin, N-cadherin, and Vimentin), the apoptosis-related proteins (cleaved caspase 3), and SOX9 of the tumors were examined by immunohistochemistry (IHC) staining. Scale bar = 100 μm. **p < 0.01, ***p < 0.001 compared to Lv-sh-NC+ antagomiR-NC group.

The effects of the ANXA2P2/miR-361-3p axis towards the growth, metastasis, and apoptosis of cervical cancer *in vivo* were evaluated on the xenograft nude mice model. Caski/DDP cells pre-transfected with lentivirus-mediated ANXA2P2 or miR-361-3p knockdown or negative control were subcutaneously injected into the armpits of nude mice, respectively. The ANXA2P2 expression level in the Lv-sh-ANXA2P2 group was markedly decreased, while observably increased in the antagomiR-361-3p group when compared to that in the control group (**Figure 7A**). Moreover, Lv-sh-ANXA2P2 notably promoted miR-361-3p expression, while antagomiR-361-3p inhibited miR-361-3p expression (**Figure 7A**). During the period of xenograft growth in nude mice, the measurement of tumor volumes was performed every 3 days. The results showed that the volumes of xenograft formed by Caski/DDP cells with the ANXA2P2 knockdown group were significantly inferior to those formed in the control group, while miR-361-3p knockdown dramatically promoted tumor volumes (**Figure 7B**). At the end of the experiment (the 22nd day), mice were euthanized and tumor tissues were excised, and the weight (**Figure 7C**) and size (**Figure 7D**) of xenografts formed by Caski/DDP cells with ANXA2P2 silencing were markedly inferior to those formed in the control group, while miR-361-3p knockdown exerted opposite effects; the effects of lncRNA ANXA2P2 knockdown were partially reversed by miR-361-3p inhibition. H&E staining was subsequently performed to examine the histopathological characteristics of the tumors; **Figure 7E** illustrates that tumor tissues in the ANXA2P2 knockdown group showed obvious necrosis, whereas miR-361-3p inhibition exacerbated the necrosis of tumor. As depicted in **Figure 7E**, IHC staining results indicated that the knockdown of ANXA2P2 observably promoted E-cadherin protein expression, while inhibiting N-cadherin and Vimentin expression, while miR-361-3p knockdown exerted opposite effects on the tumor metastasis protein expressions. Furthermore, the apoptosis-related proteins (cleaved caspase 3) and SOX9 protein were also examined by IHC staining. The silencing of ANXA2P2 significantly increased cleaved caspase 3 and reduced SOX9 proteins, while miR-361-3p inhibition notably restrained cleaved caspase 3 and facilitated SOX9 proteins (**Figure 7E**). These findings reveal the dynamic effects of the ANXA2P2/miR-361-3p axis on the growth and tumorigenesis of cervical cancer *in vivo*.

## Discussion

**Figure 8:**
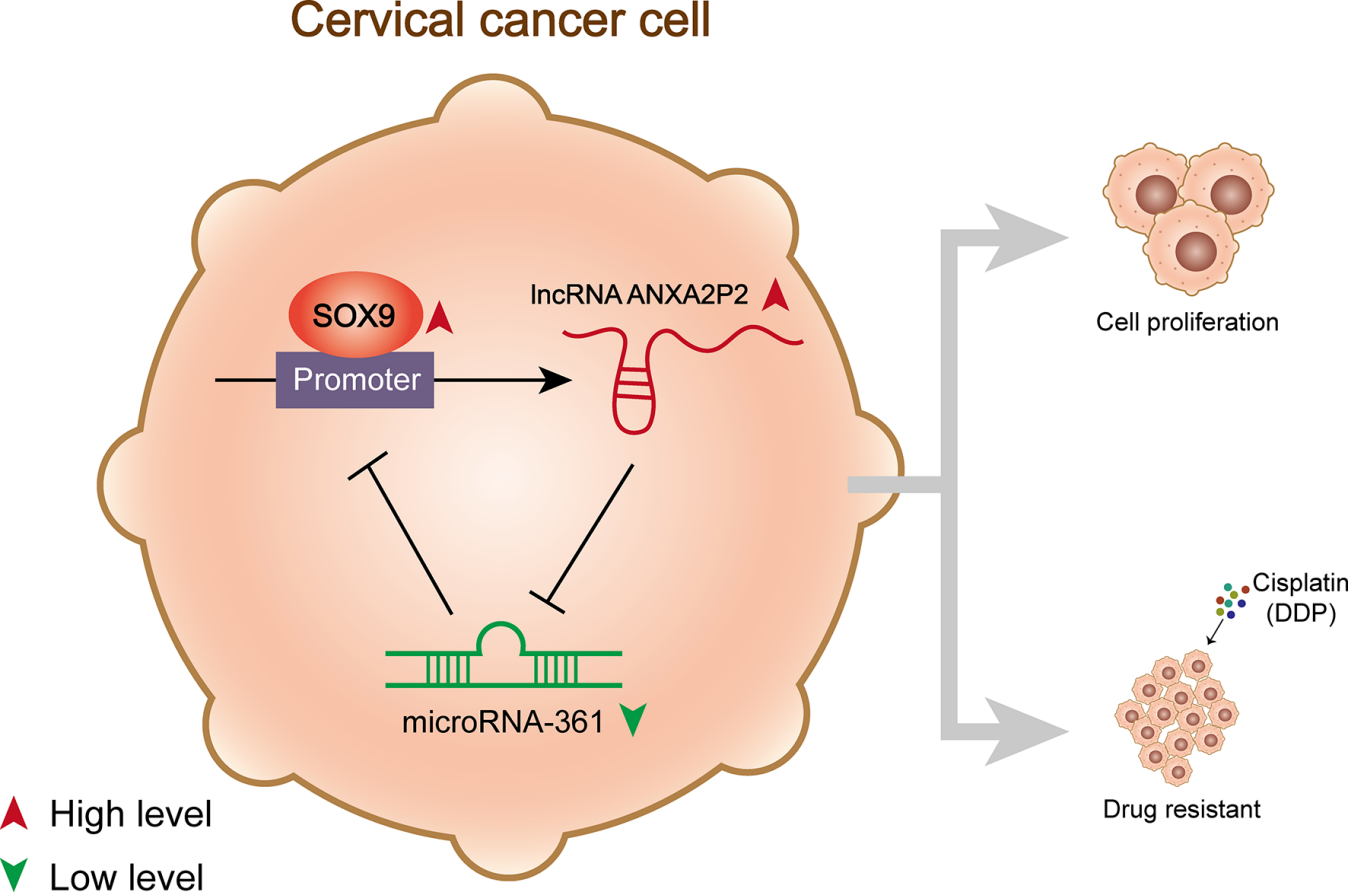
A schematic diagram showing the effects of the SOX9/lncRNA ANXA2P2/miR-361-3p/SOX9 regulatory loop on cervical cancer cell proliferation and resistance to DDP.

The specific functions of the SOX9/lncRNA ANXA2P2/miR-361-3p/SOX9 regulatory loop on the growth and DDP-resistance of cervical cancer cells were demonstrated (**Figure 8**). miR-361-3p expression was underexpressed in DDP-resistant cervical cancer cells and tissues. miR-361-3p overexpression inhibited DDP-resistant cervical cancer cell growth and resistance to DDP, whereas miR-361-3p inhibition exerted opposite effects. miR-361-3p inhibited SOX9 expression through binding; the effects of miR-361-3p inhibition on DDP-resistant cervical cancer cells were partially reversed by SOX9 knockdown. LncRNA ANXA2P2 expression was elevated in DDP-resistant cervical cancer cells and tissues. ANXA2P2 inhibited miR-361-3p expression by binding, thereby upregulating SOX9. ANXA2P2 knockdown inhibited DDP-resistant cervical cancer cell growth and resistance to DDP, whereas the effects of ANXA2P2 knockdown were partially reversed by miR-361-3p inhibition. SOX9 expression was elevated in DDP-resistant cervical cancer cells and tissues, and SOX9 activated ANXA2P2 transcription by binding.

Reportedly, miR-361-3p plays a tumor-suppressive role in multiple cancers, including non-small cancer lung cancer (13, 36–38), prostate cancer (39), thyroid cancer (16), and cervical cancer (15, 40, 41). Although previous studies have indicated the aberrant downregulation of miR-361-3p in regular cervical cancer cell lines and cervical cancer tissues (15, 40, 41), this study demonstrated the significant downregulation of miR-361-3p in DDP-resistant cervical cancer cells and tissues for the first time. Consistent with the expression trend, miR-361-3p overexpression remarkably inhibited the growth of DDP-resistant cervical cancer cells. Furthermore, miR-361-3p overexpression elevated the sensitivity of DDP-resistant cervical cancer cells to DDP under DPP stimulation. Inversely, miR-361-3p inhibition further enhanced DDP-resistant cervical cancer cells’ resistance to DDP. These data suggest that the restoration of miR-361-3p expression could potentially improve cervical cancer cell response to DDP treatment.

miRNAs commonly participate in multiple biological functions during cancer initiation and development by targeting the 3′‐UTR of mRNA and subsequent post-transcriptional regulation of gene expression. Nevertheless, miRNAs that are upregulated in cancer can possibly serve as therapeutic factors (42, 43). Therefore, inhibiting aberrantly elevated miRNAs that can promote tumorigenesis and inhibit apoptosis, or administrating tumor suppressor miRNAs could be promising therapeutic avenues in cervical cancer therapy (42, 43). Since miR-361-3p has been recognized as a tumor suppressor in cervical cancer, the downstream target of miR-361-3p was subsequently searched to investigate the mechanism underlying the tumor-suppressive role of miR-361-3p. As mentioned in our previous study, SOX9 could activate the expression of oncogenic miR-130a, enhancing cervical cancer resistance to DDP through the miR-130a/CTR1 axis (9). Moreover, SOX9 has been recognized as a direct target of miR-215-3p, which is involved in the tumor-suppressive role or miR-215-3p in cervical cancer cell (44). By using the online tool (miRWalk) and experimental validation, it was concluded that miR-361-3p directly bound to SOX9 and inhibited SOX9 expression. Consistent with the aberrant upregulation of SOX9 in DDP-resistant cervical cancer cells and tissues, SOX9 knockdown inhibited the growth of DDP-resistant cancer cells and elevated the sensitivity of DDP-resistant cervical cancer cells to DDP. Moreover, SOX9 knockdown partially reversed the effects of miR-361-3p inhibition, suggesting that miR-361-3p exerts its effects through the targeting of SOX9.

LncRNAs serve as sponges for miRNAs, competing with miRNA targets, inhibiting miRNA functions, and relieving miRNA-mediated suppression on targets (45, 46). Considering the downregulation of miR-361-3p in DDP-resistant cells and tissues, it was hypothesized that some lncRNAs might lead to miR-361-3p downregulation. ANXA2P2 has been reported as an oncogenic lncRNA, with higher expression in glioma and association with glioma progression or poorer prognosis (32, 34, 35). It has also been reported that higher ANXA2P2 expression promotes the aggressive phenotypes of hepatocellular carcinoma (33). However, the role of ANXA2P2 in cervical cancer remains unclear. In this study, the aberrant upregulation of ANXA2P2 in DDP-resistant cervical cancer cells and tissues was confirmed. Also, through targeting, ANXA2P2 inhibited miR-361-3p expression. ANXA2P2 knockdown inhibited the growth of DDP-resistant cancer cells and elevated the sensitivity of DDP-resistant cervical cancer cells to DDP, which could be partially restored by miR-361-3p inhibition. Therefore, ANXA2P2 acts on DDP-resistant cancer cells through targeting miR-361-3p.

As per previous reports, SOX9 guides signaling implicated in tumor initiation, metastasis, cancer cell proliferation, chemo- and radio-resistance, and stem cell maintenance, thereby affecting tumorigenesis as an oncogene (47). Zhou et al. reported that the silencing of SOX9 suppressed cell growth, stemness, migration, and invasion in colorectal cancer (48). Chen et al. suggested that SOX9 promotes cell stemness and tumor growth *in vitro* and *in vivo* in osteosarcoma (49). It was demonstrated that SOX9 activates oncogenic miR-130a by binding to the promoter region of miR-130a (9). Notably, herein, ChIP-Atlas database and JASPAR tool indicated the potential binding between SOX9 and ANXA2P2, which was then substantiated by experimental analyses. SOX9 activated ANXA2P2 expression by targeting its promoter. Thus, SOX9, ANXA2P2, and miR-361-3p form a regulatory loop, modulating DDP-resistant cervical cancer cell growth and response to DDP treatment (**Figure 8**).

## Data Availability Statement

Publicly available datasets were analyzed in this study. These data can be found here: TCGA-CESC data (https://portal.gdc.cancer.gov/projects/TCGA-CESC).

## Ethics Statement

The studies involving human participants were reviewed and approved by the Ethics Committee of Second Xiangya Hospital. The patients/participants provided their written informed consent to participate in this study. The animal study was reviewed and approved by the Animal Ethics Committee of Second Xiangya Hospital.

## Author Contributions

SH and YF contributed to the experimental design and oversaw the whole experimental process. WZ, JW, GL, and WX were involved in conducting the experiments. YX, J-AM, and XL. All authors contributed to the article and approved the submitted version.

## Funding

This study was supported by the National Natural Science Foundation of China (Grant nos. 81802620, 81702582, 81772928, and 81802476) and the Natural Science Foundation of Hunan Province, China (Grant no. 2021JJ30929).

## Conflict of Interest

The authors declare that the research was conducted in the absence of any commercial or financial relationships that could be construed as a potential conflict of interest.

## Publisher’s Note

All claims expressed in this article are solely those of the authors and do not necessarily represent those of their affiliated organizations, or those of the publisher, the editors and the reviewers. Any product that may be evaluated in this article, or claim that may be made by its manufacturer, is not guaranteed or endorsed by the publisher.

## Supplementary Material

Supplementary Figure 1 Differentially expressed miRNAs were screened based on TCGA-CESC data. **(A, B)** Differentially expressed miRNAs in 167 chemotherapy complete response patients compared with 27 progressive disease patients based on TCGA-CESC cervical cancer data screened out by microarray analysis and visualized in a volcano plot **(A)** and heat-map **(B)**. **(C)** The expressions of five significantly down-regulated miRNAs (hsa-miR-1269a, hsa-miR-16-2-3p, hsa-miR-660-5p, hsa-miR-361-3p, and hsa-miR-616-5p) in original Caski and HeLa cells and DDP-resistant Caski/DDP and HeLa/DDP cells were determined using qRT-PCR. **(D)** miR-361-3p expression was detected in progressive disease patients and chemotherapy complete response patients based on TCGA-CESC data.

Supplementary Figure 2 Survival analysis for miR-361-3p related lncRNAs within cervical cancer patients. **(A)** The correlation of the expression of eight lncRNAs (UBE2Q2P1, SNORA8, C15orf54, ANXA2P2, OR10A6, SCARNA8, DLEU1, and CDR1) with the survival probability of cervical cancer patients was analyzed using Kaplan–Meier plotter (https://kmplot.com/analysis/) based on TCGA data from Pan-Cancer Atlas. **(B)** The correlation of the expression of seven lncRNAs (UBE2Q2P1, SFTA1P, DLEU1, SCARNA8, ANXA2P2, SLC7A5P1, and SNORA8) with the survival probability of cervical cancer patients were analyzed using LinkedOmics (http://www.linkedomics.org/login.php).
