# Supplementary figures and images for: The Role of the SOX9/lncRNA ANXA2P2/miR-361-3p/SOX9 Regulatory Loop in Cervical Cancer Cell Growth and Resistance to Cisplatin
Source: Front Oncol. 2022 Jan 10;11:784525. doi: 10.3389/fonc.2021.784525 (PMC8784813; doi:10.3389/fonc.2021.784525)

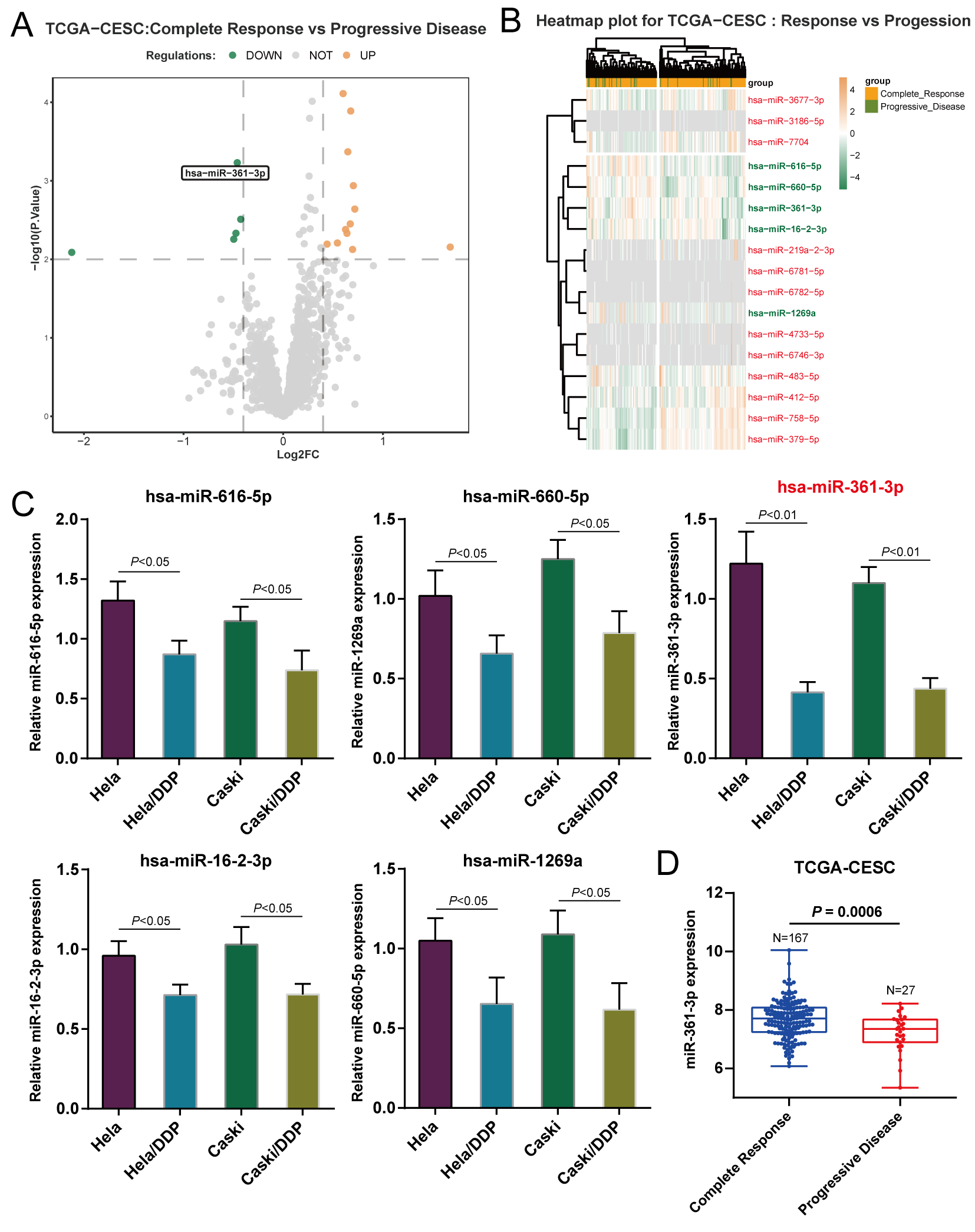

Supplement: Supplementary Figure 1 — Differentially expressed miRNAs were screened based on TCGA-CESC data. (A, B) Differentially expressed miRNAs in 167 chemotherapy complete response patients compared with 27 progressive disease patients based on TCGA-CESC cervical cancer data screened out by microarray analysis and visualized in a volcano plot (A) and heat-map (B). (C) The expressions of five significantly down-regulated miRNAs (hsa-miR-1269a, hsa-miR-16-2-3p, hsa-miR-660-5p, hsa-miR-361-3p, and hsa-miR-616-5p) in original Caski and HeLa cells and DDP-resistant Caski/DDP and HeLa/DDP cells were determined using qRT-PCR. (D) miR-361-3p expression was detected in progressive disease patients and chemotherapy complete response patients based on TCGA-CESC data. [file Image_1.tif]

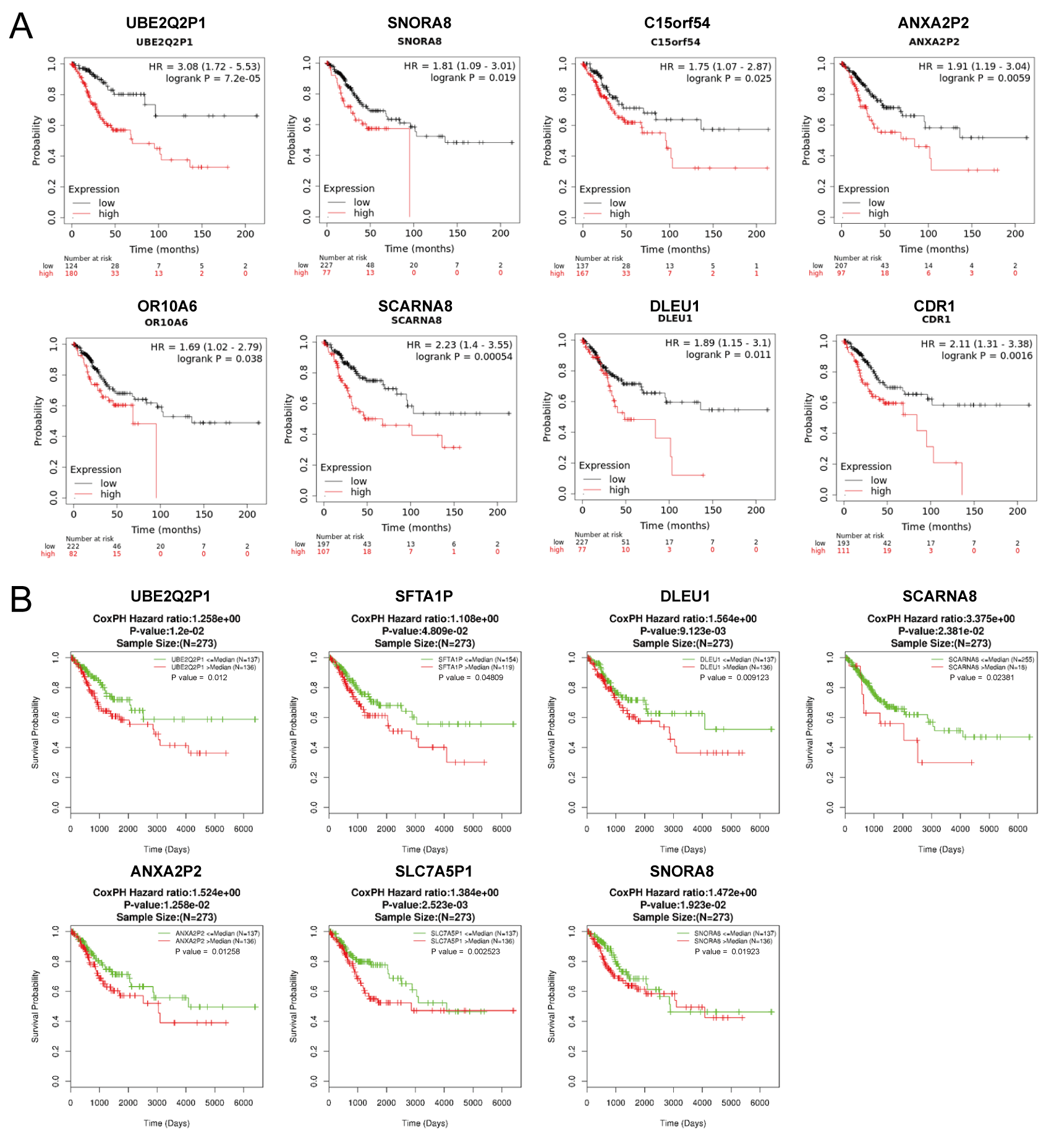

Supplement: Supplementary Figure 2 — Survival analysis for miR-361-3p related lncRNAs within cervical cancer patients. (A) The correlation of the expression of eight lncRNAs (UBE2Q2P1, SNORA8, C15orf54, ANXA2P2, OR10A6, SCARNA8, DLEU1, and CDR1) with the survival probability of cervical cancer patients was analyzed using Kaplan–Meier plotter (https://kmplot.com/analysis/) based on TCGA data from Pan-Cancer Atlas. (B) The correlation of the expression of seven lncRNAs (UBE2Q2P1, SFTA1P, DLEU1, SCARNA8, ANXA2P2, SLC7A5P1, and SNORA8) with the survival probability of cervical cancer patients were analyzed using LinkedOmics (http://www.linkedomics.org/login.php). [file Image_2.tif]
