# Supplementary material for: The Role of the SOX9/lncRNA ANXA2P2/miR-361-3p/SOX9 Regulatory Loop in Cervical Cancer Cell Growth and Resistance to Cisplatin
Source: Front Oncol. 2022 Jan 10;11:784525. doi: 10.3389/fonc.2021.784525 (PMC8784813; doi:10.3389/fonc.2021.784525)
Supplement: Supplementary file 3 [file Table_1.docx]

**Table S1. The primer sequences.**

| **Real time-PCR** | **Forward** | **Reverse** |
| --- | --- | --- |
| miR-361-3p | RT: GTCGTATCCAGTGCGTGTCGTGGAGTCGGCAATTGCACTGGATACGACAAATCA  F：TCCCCCAGGTGTGATTC | R: CAGTGCGTGTCGTGGA |
| miR-16-2-3p | RT: GTCGTATCCAGTGCGTGTCGTGGAGTCGGCAATTGCACTGGATACGACTAAAGC  F：GCGCCAATATTACTGTGCT | R: CAGTGCGTGTCGTGGA |
| miR-1269a | RT: GTCGTATCCAGTGCGTGTCGTGGAGTCGGCAATTGCACTGGATACGACCCAGTA  F：CTGGACTGAGCCGTGC | R: CAGTGCGTGTCGTGGA |
| miR-660-5p | RT: GTCGTATCCAGTGCGTGTCGTGGAGTCGGCAATTGCACTGGATACGACCAACTC  F：GCTACCCATTGCATATCG | R: CAGTGCGTGTCGTGGA |
| miR-616-5p | RT: GTCGTATCCAGTGCGTGTCGTGGAGTCGGCAATTGCACTGGATACGACAAGTCA  F：GCCACTCAAAACCCTTCAG | R: CAGTGCGTGTCGTGGA |
| DLEU1 | GAAAACCAAGCCAAACTCCATC | GCATACCAATAGTCAACTGGCAAC |
| SOX9 | AGCGAACGCACATCAAGAC | CTGTAGGCGATCTGTTGGGG |
| ANXA2P2 | GAAGGACATTATTTCGGACACATCT | CTCTGCTCTTCTACCCTTTGCC |
| ANXA2P2 promoter | CTCAACATCATCCCCTCCTACCCT | CTCCGCATCAACTCTGTCCACACT |
| GAPDH | CTCAGACGGCAGGTCAGGTCCACC | CCACCCATGGCAAATTCCATGGCA |
| U6 | CTCGCTTCGGCAGCACA | AACGCTTCACGAATTTGCGT |
| **Transfection RNA** |  |  |
| mimics NC | UUCUCCGAACGUGUCACGUTT | ACGUGACACGUUCGGAGAATT |
| inhibitor NC | UUCUCCGAACGUGUCACGUTT |  |
| miR-361 mimics | TCCCCCAGGTGTGATTCTGATTT | ATCAGAATCACACCTGGGGGAUU |
| miR-361 inhibitor | AAATCAGAATCACACCTGGGGGA |  |
| si-NC | UUCUCCGAACGUGUCACGUTT | ACGUGACACGUUCGGAGAATT |
| si-ANXA2P2 | GAUAGGUACAAGAGUUACATT | UGUAACUCUUGUACCUAUCTT |
| siRNA1-SOX9 | GGGAGUAAACAAUAGUCUATT | UAGACUAUUGUUUACUCCCTT |
| siRNA2-SOX9 | GUGUGAUCAGUGUGCUAAATT | UUUAGCACACUGAUCACACTT |
| siRNA3-SOX9 | AGAUACUUCUGUAACUUAATT | UUAAGUUACAGAAGUAUCUTT |
| **Plasmid construction** |  |  |
| wt-SOX9 3’UTR | aattctaggcgatcgctcgagAAACTACTCTTAGTTGAACAGTGTGCC | attttattgcggccagcggccgcTTCTCTTCTTTAAAAAATATATATATGATAAAGCT |
| mut-SOX9 3’UTR | GTGATCAGTTTgtgaggTTAACTTTGCTTAATTCCTCAGGCT | AcctcacAAACTGATCACATAACACAATTCTGTTT |
| ANXA2P2 promoter-wt | gcgtgctagcccgggctcgagATTCCTTGGCCTCAACATCATC | cagtaccggaatgccaagcttCCAGCTAAACTACTGCTTGCCTG |
| ANXA2P2 promoter-mut | CCTGagcggtgagTTAATGACTCTAGCTCAAAATAATCCTTAT | TTAActcaccgctCAGGTATAAGAGTACTCTGACCCTTCC |
