# Supplementary material for: The Role of the SOX9/lncRNA ANXA2P2/miR-361-3p/SOX9 Regulatory Loop in Cervical Cancer Cell Growth and Resistance to Cisplatin
Source: Front Oncol. 2022 Jan 10;11:784525. doi: 10.3389/fonc.2021.784525 (PMC8784813; doi:10.3389/fonc.2021.784525)
Supplement: Supplementary file 4 [file Table_2.docx]

| **logFC** | **AveExpr** | **t** | **P.Value** | **adj.P.Val** | **B** | **MIRBASE_ID** | **miRNA** | **change** |
| --- | --- | --- | --- | --- | --- | --- | --- | --- |
| -2.12168 | 2.438893 | -2.68759 | 0.008158 | 0.461598 | -2.76591 | MIMAT0005923 | hsa-miR-1269a | DOWN |
| -0.4964 | 4.383461 | -2.8049 | 0.005547 | 0.423034 | -2.62001 | MIMAT0004518 | hsa-miR-16-2-3p | DOWN |
| -0.47441 | 5.493888 | -2.86314 | 0.004657 | 0.384366 | -2.46534 | MIMAT0003338 | hsa-miR-660-5p | DOWN |
| -0.46129 | 7.606058 | -3.49478 | 0.000588 | 0.171956 | -0.60421 | MIMAT0004682 | hsa-miR-361-3p | DOWN |
| -0.42643 | 1.446997 | -2.99824 | 0.003094 | 0.361823 | -2.07339 | MIMAT0003284 | hsa-miR-616-5p | DOWN |
| 0.439813 | 0.318087 | 3.007015 | 0.006406 | 0.432144 | -2.34719 | MIMAT0027393 | hsa-miR-6746-3p | UP |
| 0.543387 | 1.096113 | 2.775532 | 0.006197 | 0.432144 | -2.59076 | MIMAT0030019 | hsa-miR-7704 | UP |
| 0.600568 | 0.329375 | 4.671164 | 7.75E-05 | 0.070136 | -0.77686 | MIMAT0019857 | hsa-miR-4733-5p | UP |
| 0.625193 | 0.534702 | 3.399103 | 0.004172 | 0.384366 | -1.92114 | MIMAT0015067 | hsa-miR-3186-5p | UP |
| 0.639114 | 2.367758 | 2.862538 | 0.004668 | 0.384366 | -2.45513 | MIMAT0018101 | hsa-miR-3677-3p | UP |
| 0.648014 | 1.211798 | 3.591503 | 0.000427 | 0.149907 | -0.29346 | MIMAT0004761 | hsa-miR-483-5p | UP |
| 0.672483 | 0.331286 | 4.49081 | 0.003541 | 0.384366 | -2.94746 | MIMAT0027462 | hsa-miR-6781-5p | UP |
| 0.676813 | 0.306089 | 6.650182 | 0.000129 | 0.070136 | -1.94205 | MIMAT0027464 | hsa-miR-6782-5p | UP |
| 0.695491 | 8.812976 | 2.702564 | 0.007493 | 0.43808 | -2.8847 | MIMAT0000733 | hsa-miR-379-5p | UP |
| 0.703427 | 2.242171 | 3.300198 | 0.001153 | 0.252883 | -1.21377 | MIMAT0022929 | hsa-miR-758-5p | UP |
| 0.718029 | 2.013678 | 3.093237 | 0.002291 | 0.32121 | -1.81669 | MIMAT0026557 | hsa-miR-412-5p | UP |
| 1.67494 | 1.063006 | 2.810151 | 0.006989 | 0.43808 | -2.39256 | MIMAT0004675 | hsa-miR-219a-2-3p | UP |

**Table S2. Differentially expressed miRNAs screen from TCGA-CESC dataset (Complete Response Patients vs Progressive Disease Patients)**
