# Supplementary material for: The Role of the SOX9/lncRNA ANXA2P2/miR-361-3p/SOX9 Regulatory Loop in Cervical Cancer Cell Growth and Resistance to Cisplatin
Source: Front Oncol. 2022 Jan 10;11:784525. doi: 10.3389/fonc.2021.784525 (PMC8784813; doi:10.3389/fonc.2021.784525)
Supplement: Supplementary file 5 [file Table_3.docx]

**Table S3. LncRNAs significantly negatively correlated with the expression of miR-361-3p based on** **TCGA-CESC database**

| **ncRNA** | **r value** | **p value** |
| --- | --- | --- |
| FLJ16779 | -0.15421 | 0.007866 |
| UBE2Q2P1 | -0.19273 | 0.000859 |
| PGM5P2 | -0.16747 | 0.003858 |
| SFTA1P | -0.18242 | 0.001623 |
| SNORA8 | -0.15083 | 0.009352 |
| KLKP1 | -0.1945 | 0.000767 |
| SLC7A5P1 | -0.19076 | 0.000973 |
| C15orf54 | -0.19062 | 0.000981 |
| ANXA2P1 | -0.15077 | 0.010377 |
| ANXA2P2 | -0.15001 | 0.012508 |
| SNHG4 | -0.20785 | 0.000318 |
| C1orf229 | -0.1913 | 0.00094 |
| OR10A6 | -0.18519 | 0.001372 |
| SCARNA8 | -0.19446 | 0.000769 |
| DLEU1 | -0.1778 | 0.002137 |
| CDR1 | -0.18165 | 0.0017 |

Note: TCGA-CESC: The Cancer Genome Atlas (TCGA) cervical squamous cell carcinoma and endocervical adenocarcinoma (CESC).
